# Mechanical force increases tooth movement and promotes remodeling of alveolar bone defects augmented with bovine bone mineral

**DOI:** 10.1186/s40510-023-00501-3

**Published:** 2024-01-08

**Authors:** Jie Deng, Zi-Meng Zhuang, Xiao Xu, Bing Han, Guang-Ying Song, Tian-Min Xu

**Affiliations:** 1 Department of Orthodontics, Peking University School and Hospital of Stomatology & National Center of Stomatology & National Clinical Research Center for Oral Diseases & National Engineering Laboratory for Digital and Material Technology of Stomatology & Beijing Key Laboratory for Digital Stomatology & Research Center of Engineering and Technology for Computerized Dentistry Ministry of Health, 22 Zhongguancun South Avenue, Haidian District, Beijing, 100081 People’s Republic of China; 2 Department of Orthodontics, Nanjing Stomatological Hospital, Affiliated Hospital of Medical School, Nanjing University, No. 30 Zhongyang Road, Nanjing, 210008 People’s Republic of China; 3 Department of Periodontology, Peking University School and Hospital of Stomatology & National Center of Stomatology & National Clinical Research Center for Oral Diseases & National Engineering Laboratory for Digital and Material Technology of Stomatology & Beijing Key Laboratory for Digital Stomatology & Research Center of Engineering and Technology for Computerized Dentistry Ministry of Health, 22 Zhongguancun South Avenue, Haidian District, Beijing, 100081 People’s Republic of China

**Keywords:** Orthodontic tooth movement, Mechanical force, Bovine bone mineral grafting, Bone defects, Macrophage polarization, Phagocytosis

## Abstract

**Background:**

Orthodontic tooth movement (OTM) in a region containing alveolar bone defects with insufficient height and width is hard to achieve. Bovine bone mineral (Bio-Oss) is available to restore the alveolar defect; however, whether the region augmented with a bovine bone mineral graft (BG) is feasible for OTM, and the mechanisms by which macrophages remodel the BG material, is uncertain under the mechanical force induced by OTM.

**Material and methods:**

Rats were divided into three groups: OTM (O), OTM + BG material (O + B), and Control (C). First molars were extracted to create bone defects in the O and O + B groups with bovine bone mineral grafting in the latter. Second molars received OTM towards the bone defects in both groups. After 28 days, maxillae were analyzed using microfocus-computed tomography (μCT) and scanning-electron-microscopy (SEM); and macrophages (M1/M2) were stained using immunofluorescence. THP-1 cell-induced macrophages were cultured under mechanical force (F), BG material (B), or both (F + B). Phagocytosis-related signaling molecules (cAMP/PKA/RAC1) were analyzed, and conditioned media was analyzed for MMP-9 and cytokines (IL-1β, IL-4).

**Results:**

Our study demonstrated that alveolar defects grafted with BG materials are feasible for OTM, with significantly increased OTM distance, bone volume, and trabecular thickness in this region. SEM observation revealed that the grafts served as a scaffold for cells to migrate and remodel the BG materials in the defect during OTM. Moreover, the population of M2 macrophages increased markedly both in vivo and in cell culture, with enhanced phagocytosis via the cAMP/PKA/RAC1 pathway in response to mechanical force in combination with BG particles. By contrast, M1 macrophage populations were decreased under the same circumstances. In addition, M2 macrophage polarization was also indicated by elevated IL-4 levels, reduced IL-1β levels, and less active MMP-9 in cell culture.

**Conclusion:**

This study explored the mechanisms of mechanical force-induced alveolar bone remodeling with bovine bone mineral grafts during OTM. The results might provide molecular insights into the related clinical problems of whether we can move teeth into the grafted materials; and how these materials become biologically remodeled and degraded under mechanical force.

## Introduction

Orthodontic tooth movement (OTM) in a region of alveolar bone defects with insufficient height and buccal-lingual width can be harmful to patients [1]. If patients with alveolar defects require orthodontic treatment, bone augmentation before OTM is usually necessary [2]. In recent years, deproteinized bovine-derived bone mineral (Bio-Oss) has become available for periodontal regeneration and reconstructive surgery [2]. However, the feasibility and safety of OTM into a region of bone defects augmented with bovine bone mineral grafts (BG) are controversial. In addition, the underlying mechanisms of mechanical force-induced alveolar bone remodeling augmented with BG materials remain unclear.

Force-induced alveolar bone remodeling is a unique process that is involved in tooth development, movement, eruption, and mastication [3]. In a previous study, we found that orthodontic/mechanical force-induced remodeling on the alveolar bone of rats was characterized by the infiltration of many osteoclasts and inflammatory cells into the remodeled areas at the beginning of OTM [4–6]. Notably, that macrophages are the major infiltrating cells that respond rapidly to biomaterial grafts in both soft and hard tissues [7]. These cells fuse to multinucleated giant cells and their morphological variants, remaining at the biomaterial-tissue interfaces for their lifetime in vivo [7].

In addition, a critical function of macrophages is to mediate the remodeling of the biomaterials involved in OTM through extracellular degradation and phagocytosis [8, 9]. During phagocytosis, reshaping of the macrophage membrane leads to full engulfment of the bio-particles and their release in cytoplasmic organelles, named phagosomes [10]. After maturation, lysosomes and other hydrolytic enzymes are merged into phagosomes to dissolve, digest, and degrade the internalized bio-particles [7]. This phagocytic process is mediated by the increased production of cyclic adenosine monophosphate (cAMP), which results in protein kinase A (PKA)-dependent augmentation of Ras-related C3 botulinum toxin substrate 1 (RAC1) activity [11].

Moreover, macrophages are highly plastic cells that can be polarized to different phenotypes in response to various microenvironmental signals [12]. Originally, two subpopulations were identified, comprising the classically-activated macrophages or M1 phenotype, and the alternatively-activated macrophages or M2 phenotype. M1 macrophages have pro-inflammatory functions that are vital for immune defense and the destruction of invading pathogens. However, polarized M2 macrophages dramatically increase phagocytic activities and have anti-inflammatory functions that promote the resolution of inflammation and tissue repair [12]. Studies suggested that during OTM, the mechanical force induces macrophage infiltration and their polarization to the M1-like phenotype [13, 14]. Moreover, increasing the ratio of M2 to M1 macrophages accelerates the regeneration and reconstitution of bone tissue [15, 16]. However, the molecular mechanisms regarding mechanical force-induced alveolar bone remodeling with grafted biomaterials during OTM remain unknown.

Therefore, this study aimed to investigate the biological effects of OTM on bone defects that have been grafted with BG materials. We used both rat and cell culture models to explore: (a) The feasibility of OTM in the bovine bone mineral grafted region, (b) the morphological changes of alveolar bone defect remodeling with or without bovine bone mineral grafts during OTM, and (c) the roles of macrophages during this remodeling process. From a clinical aspect, this study is expected to provide molecular insights into the clinical problem of moving teeth into bone grafting materials, as well as how the grafting materials undergo biological remodeling and degradation under mechanical force.

## Materials and methods

### Animals

Twenty-one male SD rats (body weight/age: 151 g/42 days) were purchased from Beijing Vital River (Beijing, China). All rats were housed in the animal room of Peking University School and Hospital of Stomatology, with care provided by the center’s personnel. All procedures were conducted at the same location. The protocols for the animal studies were approved by the Peking University Biomedical Ethics Committee (approval number: LA2020515). The Three Rs (replacement, reduction, and refinement) were reviewed by the Committee and approved. All animal experiments were conducted in strict adherence to the guidelines outlined in ARRIVE 2.0 [17] for ethical animal research.

### Establishment of a rat OTM model with alveolar bone defects and BG material augmentation

**Fig. 1:**
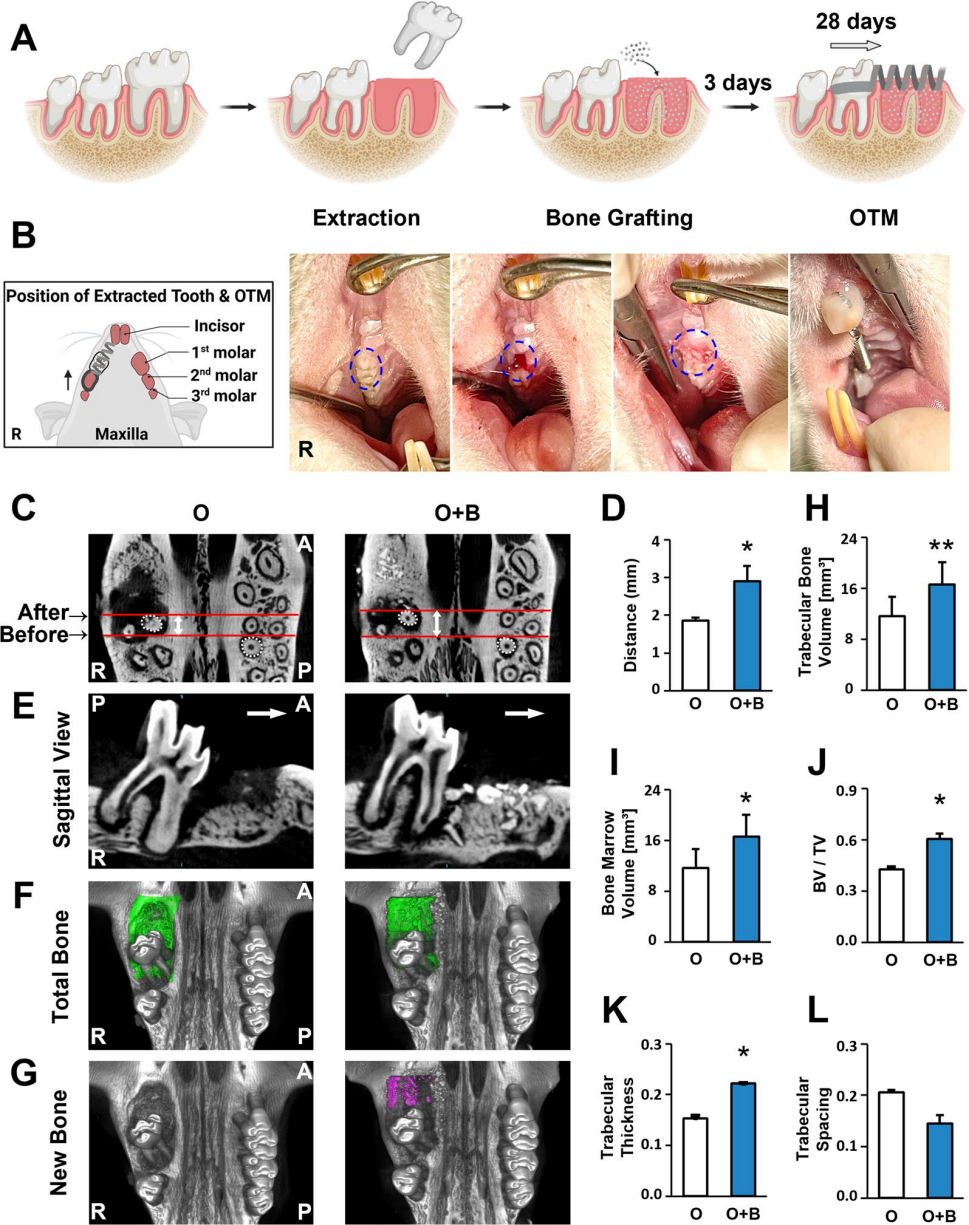
Establishment of a rat orthodontic tooth movement (OTM) model with alveolar bone defects and grafted using BG augmentation. **A** The BG materials were grafted immediately after tooth extraction (the 1st molar). After 3 days, tooth movement was performed on the 2^nd^ molar and continued for 28 days. **B** A schematic diagram from the occlusive view of the maxilla, indicating the location of alveolar defect generated by the extraction of the 1st molar and grafted with BG materials on the right side. Occlusive view of a rat receiving OTM on the 2nd molar. **C** μCT reconstruction of the maxilla. Red lines indicate the distance of tooth movement on the 2nd molar. R: right side. A: anterior. P: posterior. O: the OTM group. O + B: the OTM + BG material group. **D** Quantitative analysis of the distance of the tooth movement on the 2nd molar. **E** Sagittal view of the tooth movement of the 2nd molar. **F** μCT reconstruction of the current total bone volume (indicated in green) in the alveolar defect region with/without BG materials during OTM. **G** The new bone volume is indicated in purple. **H–L** Quantitative analysis of the trabecular bone volume, bone marrow volume, bone volume fraction (BV/TV), trabecular thickness, and trabecular spacing. Each value represents the mean (n = 7/group) ± the standard error. * *p* < 0.05, ** *p* < 0.01

Twenty-one rats were divided into three groups: OTM (O), OTM + BG material (O + B), and Control (C), with 7 rats/per group. The alveolar bone defects were generated by molar extraction followed by augmentation with BG materials. Specifically, fourteen rats in the O and O + B groups were subjected to general anesthesia using 1% pentobarbital sodium [intraperitoneally (i.p.), 50–60 mg/kg]. After anesthesia, the first molars on the right maxillae were extracted to create alveolar bone defects. In the O + B group, the extracted sockets were filled with BG material (Bio-Oss™, 0.25–1 mm, Geistlich Pharma North America Inc., Princeton, NJ, USA) immediately and the wounds were sutured using a 6–0 absorbable thread. In the O group, only the first molars were extracted without bovine bone mineral grafting. These two groups of rats were allowed to recover from surgery for 3 days. For the C group, seven rats were used as normal controls. The procedures described above are illustrated in Fig. 1A.

After three days, when the acute inflammation had subsided, OTM was commenced by moving the second maxillary right molar towards the mesial side using a custom-made orthodontic nickel–titanium coil (0.2 mm wire diameter, 1 mm loop diameter, 6.5 mm length), while the central incisors served as anchorages. The nickel-titanium coil was fixed at both ends of the incisor and the second molar using stainless steel ligature wires (0.2 mm diameter), and the ligature wires were reinforced with light-curing resin to prevent slippage. An orthodontic force dynamometer (Gauge: 0–400 g/0–16Ounce, AcmeDent, Concord, Canada) was used to measure 25 g of force generated by the orthodontic nickel-titanium coil for OTM in each rat. Thus, a rat model of OTM with/without bovine bone mineral grafting in the alveolar bone defects was established (Fig. 1B). The duration of OTM was 28 days for both the O and O + B groups. At 28 days, carbon dioxide inhalation was used to euthanize the rats for sample collection and further analysis.

### Three-dimensional μCT morphometric analysis of the bovine bone mineral grafting area after OTM

After euthanasia, the maxillae were collected and fixed in 4% paraformaldehyde for 48 h. Then microfocus computed tomography [micro-CT (μCT), SkyScan 1174, Bruker, Billerica, MA, USA] was used to scan the maxillary samples followed by a three-dimensional (3D) reconstruction. The distance of OTM was measured as the relative anterior–posterior distance between the mesial-palatal root of the right second molar compared with the mesial-palatal root of the contralateral (left) second molar (the distance of OTM was illustrated between the red lines in Fig. 1C). The relative newly-formed bone volume, trabecular bone volume (mm^3^), bone marrow volume (mm^3^), bone volume fraction (BV/TV), trabecular thickness, and trabecular spacing were assessed quantitatively using μCT analysis.

### Scanning electron microscopy

The alveolar bone samples (including teeth) were fixed using a 2.5% glutaraldehyde fixative solution (Solarbio, P1126, Beijing, China) for scanning electron microscopy (SEM) at 4 °C for 48 h. To maintain the integrity of the BG materials, no decalcification was performed. The alveolar bone samples of each group were dissected sagittally using a sliding microtome (Leica, Shanghai, China) for hard tissue. After dehydration using gradient alcohol and vacuum drying, the samples were subjected to SEM observation and analysis.

### Immunofluorescence

Immunofluorescence (IF) was used to observe the polarization of M1/M2 macrophages in the bovine bone mineral grafting area with mechanical force-induced bone remodeling during OTM. The alveolar bone samples from the C, O, and O + B groups (including teeth) were fixed in 4% paraformaldehyde for 48. Then, the samples were decalcified using ethylenediaminetetraacetic acid (EDTA), followed by dehydration in gradient alcohol and clearing in xylene. After embedding in paraffin, serial Sects. (5 μm thick) were acquired, mounted on slides, and stained using IF. To detect macrophage polarization, antibodies recognizing CD11b (integrin subunit alpha M) (1:1000, ab133357, Abcam, Cambridge, UK) as a cell surface marker for M0 macrophages were used. Antibodies recognizing CD68 (1:50, sc-20060, Santa Cruz Biotechnology, Santa Cruz, CA, USA) were used to label the M1 macrophages (CD68^+^CD11b^+^). Antibodies recognizing CD206 (1:10,000, 60,143-1-Ig, Proteintech, Rosemont, IL, USA) were used to label the M2 macrophages (CD206^+^CD11b^+^). The M1/M2 polarization of macrophages in the OTM area with bovine bone mineral grafts was observed [18]. Five random fields of view were analyzed using Image J software (NIH, Bethesda, MD, USA).

### Cell culture study

Human THP-1 monocytes were cultured in Roswell Park Memorial Institute (RPMI) 1640 medium (Gibco™, Grand Island, NY, USA) in 6-well plates. Each well contained 5 × 10^6^ cells/mL, supplemented with 5% fetal bovine serum and 100 units/mL penicillin, and 100 μg/mL streptomycin. The cells were cultured in a humidified atmosphere of 5% CO_2_ and 95% air at 37 °C. In addition, 20 ng/mL of macrophage colony-stimulating factor (M-CSF) (PeproTech, Rocky Hill, NJ, USA) and 200 ng/mL of phorbol 12-myristate 13-acetate (PMA) (P1585, Sigma-Aldrich, St. Louis, MO, USA) were added to the cells and cultured for 3 days to induce differentiation of monocytes into macrophages, as described previously [19]. Other cell culture reagents and chemical reagents were purchased from Thermo Fisher Scientific (Waltham, MA, USA).

Then, the THP-1-induced macrophages were randomly distributed into four groups: Control (C), BG material (B), Force (F), and Force + BG material (F + B) groups. For the B and F + B groups, 1 mg/mL of BG material was added to the culture media. The compressive force was loaded onto the F and F + B groups. The static compressive force was loaded onto the induced macrophages as described previously [20]. The cells were subjected to 1 g/cm^2^ of continuous compressive force for 0, 6, and 24 h. The C group was treated without BG material or Force (Fig. 4A).

At 0, 6, and 24 h time points, morphometric analysis was carried out under light microscopy. The protein and RNA expression levels of signaling molecules (PKA/RAC1) associated with phagocytosis were measured using western blotting and quantitative real-time reverse transcription polymerase chain reaction (qRT-PCR). The cell supernatants were collected for enzyme-linked immunosorbent assays (ELISAs) and gelatin zymogram analyses.

### Western blotting (WB) analysis

At 0, 6, and 24 h, the induced macrophages in each group were treated with EDTA-free radioimmunoprecipitation assay (RIPA) buffer (pH 7.0) (Sigma-Aldrich) containing a Halt™ protease and phosphatase inhibitor cocktail (1:100, 78,441, Thermo Scientific). The total protein in each group of cells was extracted by centrifugation after complete cell lysis, and the protein concentrations were determined using a bicinchoninic acid (BCA) protein assay kit (Thermo Fisher Scientific). According to the concentrations of proteins, samples were subjected to sodium dodecyl sulfate–polyacrylamide gel electrophoresis (SDS-PAGE) (8% separating and 4% stacking gels) and then electrophoretically transferred onto nitrocellulose membranes (Millipore, Billerica, MA, USA). After transfer, the membranes were blocked using 5% bovine serum albumin [BSA, diluted with tris-buffered saline-Tween20 (TBST)] for 1 h, and then the diluted primary antibodies were added and incubated overnight at 4 °C. The primary antibodies recognized PKA (1:200, sc-28315, Santa Cruz, Biotechnology), and RAC1 (1:200, sc-514583, Santa Cruz, Biotechnology). β-actin was detected as the internal control. After incubation with the primary antibodies, depending on the species source of the different primary antibodies, diluted secondary antibodies [goat anti-mouse IgG-HRP (1:5000, SE131Solarbio) or goat anti-rabbit IgG-horseradish peroxidase (HRP) (1:5000, SE134, Solarbio)] were added and incubated for 1 h at room temperature. The SuperSignal™ West Dura Extended Duration Substrate (34,075, Thermo Fisher Scientific) was applied to detect and visualize the immunoreactive protein bands, which were subsequently scanned and documented using the BIO-RAD ChemiDoc™ MP Imaging System (BIO-RAD, Hercules, CA, USA). The densitometric units were measured in the linear range of immunoreactivity for PKA, and RAC1 levels using Image J analysis software.

### Quantitative real-time reverse transcription polymerase chain reaction (qRT-PCR)

Total RNA was extracted from each group of induced macrophages using the TRIzol reagent (Invitrogen, Carlsbad, CA, USA) at 0, 6, and 24 h. Then, 30 μL of 1 × RNAsafe was used to lyse the RNA precipitate, and then the RNA concentration and integrity were quantified by measuring the optical density (OD) value after incubation for 60 °C for 20 min in a water bath (measured using a DU800 UV-optical spectrophotometer; Beckman Coulter, Brea, CA, USA). To reverse-transcribe the RNA to cDNA, iScript reverse transcriptase (Bio-Rad) was mixed with poly-oligo (dT), the RNA, and primers, and processed on a PCR thermal cycler (Takara, Dalian, China). Quantitative PCR was performed on an ABI Prism 7500 RT-PCR System (Biosystems, Foster City, CA, USA). Specifically, each well was loaded with 20 μL of the PCR mixture, including 10 μL of RNase-free water, 8 μL of FastStart Universal SYBR Green Master Mix (Rox), 1 μL of primer, and 1 μL of template cDNA. The cycling parameters for PCR amplification were as follows: 15 min at 95 °C; followed by 40 cycles of 15 s at 95 °C, followed by 1 h at 60 °C. The expression levels of the target genes (*Pka/Rac1*) were normalized by that of glyceraldehyde-3-phosphate dehydrogenase (*Gapdh*). The sequences of primers used in this experiment are shown in Table 1.

**Table 1:** Sequences of primers used for quantitative real-time PCR analysis

Gene	Forward primer (5'-3')	Reverse primer (5'-3')
*Pka*	CACTGCTCGACCTGAGAGAC	CCGCATCTTCCTCCGTGTAG
*Rac1*	ATGTCCGTGCAAAGTGGTATC	CTCGGATCGCTTCGTCAAACA
*Gapdh*	ACAACTTTGGTATCGTGGAAGG	GCCATCACGCCACAGTTTC

Pka, protein kinase A; Rac1, Ras-related C3 botulinum toxin substrate 1; Gapdh, glyceraldehyde-3-phosphate dehydrogenase

#### ELISA

ELISA was performed to determine the levels of interleukin-1 β (IL-1β) and interleukin-4 (IL-4) in the supernatants of each group of cells collected at 0, 6, and 24 h. ELISA kits for IL-1β (MM-0181H2, MEIMIAN, Chengdu, China) and IL-4 (MM-0051H2, MEIMIAN) were used in this experiment. All measurements were performed according to the kit manufacturer’s instructions. For cAMP, the levels in each group of induced macrophages were determined using ELISA (Cayman Chemicals).

### Gelatin zymography for matrix metalloproteinase-9 (MMP-9) analysis

At 0, 6, and 24 h, the conditioned media from each group of cells were collected and analyzed for pro- (92 kDa) and active- (82 kDa) MMP-9 using gelatin zymography, as described previously [21–24]. The clear zones of gelatin lysis against the background indicated gelatinolytic activity and were scanned densitometrically using the BIO-RAD ChemiDoc™ MP Imaging System (BIO-RAD, CA, USA). The results were analyzed using Image J to quantitatively assess the gelatinase activity [25]. The MMP-9 standard was purchased from R&D Systems, Inc. (Minneapolis, MN, USA).

### Statistical analysis

SPSS19.0 (IBM Corp., Armonk, NY, USA) was used to analyze the data. All statistical data were determined using analysis of variance (*ANOVA*), and also using Student’s *t*-test (two investigators carried out the data analysis, separately), with *p* < 0.05 taken as statistically significant. For the in vivo data, each value represented the mean (n = 7/group) ± the standard error of the mean (S.E.M.). For data from the cell culture model, each value represented the mean (n = 3/group) ± the S.E.M. The study was single-blinded. The two investigators carrying out the data analysis did not know which group received OTM or OTM + BG in the animal study; or F, BG or F + BG in the cell culture study. All experiments were independently performed at least three times.

## Results

### Tooth movement is feasible in the bone augmentation region with bovine bone mineral grafts

The μCT 3D reconstruction revealed the morphometric changes to the alveolar defects augmented with BG materials after moving the second molar to this region (Fig. 1C). The OTM distance by the second molar was significantly increased in the O + B group compared with that in the O group without grafting (Fig. 1D; *p* < 0.05). Observation from the sagittal view indicated that without support by the BG materials underneath, the second molars in the O group were moved with more of an incline towards the mesial side than those in the O + B group. However, the second molars in the O + B group were moved relatively upright towards the mesial (Fig. 1E).

### Effects of mechanical force on remodeling of bovine bone mineral grafts in the alveolar bone defect during OTM

The total bone volume in the areas of alveolar bone defects was reconstructed using μCT analysis and is indicated in Fig. 1F. The newly-formed bone volume was separated from the total bone volume and is marked out in Fig. 1G. With bovine bone mineral grafting materials, the amounts of newly-formed bone in the O + B group were markedly higher than those in the O group at 28 days (Fig. 1G). Further analysis confirmed that four parameters were significantly higher in the O + B group than in the O group: Trabecular bone volume (*p* < 0.01), bone marrow volume (*p* < 0.05), bone volume fraction (*p* < 0.05), and trabecular thickness (*p* < 0.05) (Fig. 1H–K). Interestingly, there was a trend of reduction in trabecular spacing in the O + B group compared with the O group (Fig. 1L), which exactly reflected the significantly enhanced trabecular thickness. These results revealed that mechanical force accelerated BG material remodeling with new bone formation in the alveolar bone defect during OTM.

### Ultrastructure of bovine bone mineral grafts in the alveolar bone defect under mechanical force during OTM

**Fig. 2:**
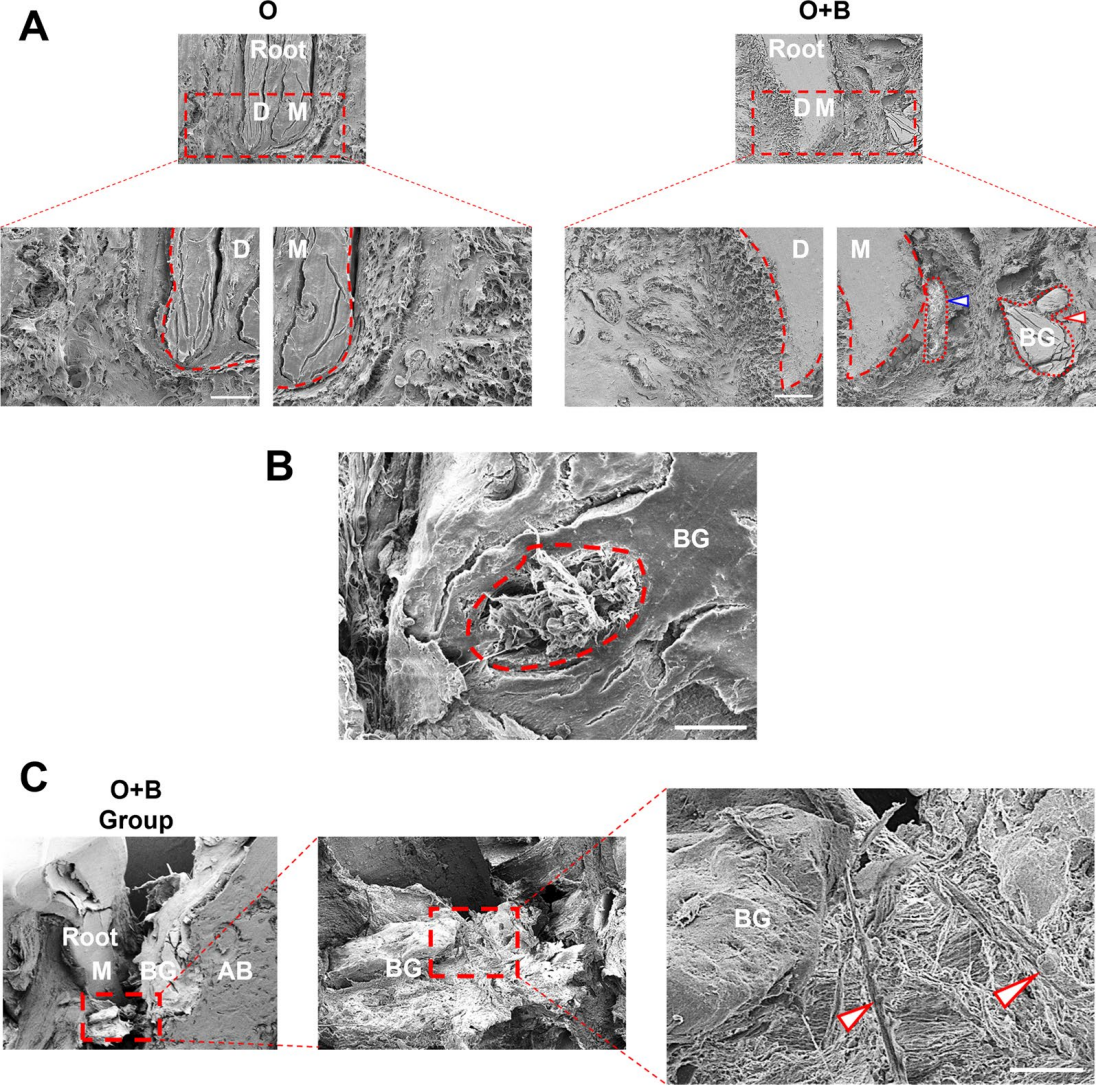
Ultrastructure of the mesial root of the 2nd molar in the alveolar defect with/without BG augmentation. **A** SEM observation of the mesial root of the second molar in the alveolar defect with/without BG augmentation (200 × magnification). The blue arrow indicates the remodeled bovine bone mineral grafts. The red arrow indicates the un-remodeled bovine bone mineral grafts. M: mesial. D: distal. BG: bovine bone mineral graft. **B** The internal ultrastructure bovine bone mineral grafted materials (600 × magnification). **C** Magnification (700×) of the alveolar defect area adjacent to the mesial surface (M) of the 2nd molar with the BG materials. M: mesial. D: distal mesial. BG: BG materials. AB: alveolar bone

SEM was used to observe the mesial root of a second molar that was moved to the alveolar defect region augmented with BG materials (Fig. 2A). At 200 × magnification, in the O + B group, the root mesial surface (M) was closely adjacent to the BG particles that were encapsulated by the alveolar bone tissues with ongoing remodeling at the mesial sides (Fig. 2A). When the root encountered the BG particles, the adjacent periodontal tissues, including the alveolar bone and periodontal ligaments, were remodeled and replaced by “fibrous bone-like tissues” with high density and a whitish appearance (Fig. 2A, indicated by the arrow). Additionally, BG particles next to the root distal surface (D) seemed to be completely degraded at the distal side after 28 days of OTM (Fig. 2A). Moreover, observations at 600× magnification further exposed the internal ultrastructure of the BG particles, indicating that the fibrous bone-like tissues were growing within the BG material (Fig. 2B). This was presumably caused by the mutual encapsulation of the periodontal tissues and bovine bone mineral grafting materials undergoing mechanical force-induced remodeling.

To clarify the ultrastructure of the fibrous bone-like tissues, we magnified the root mesial surface adjacent to the BG particles by 700× (Fig. 2C). In this area, SEM observation revealed that the bovine bone mineral grafts acted as a scaffold, with the fibrous bone-like tissues connected in between. Intriguingly, cells were observed to be "climbing" on the fibrous bone-like tissues and migrating to the bone defect region (Fig. 2C, indicated by the arrow). We inferred that the cells that migrated to the defect region might play a crucial role in BG material remodeling during OTM (Appendix Fig. 6). Notably, BG particles serving as a scaffold was also important for bone deposition and new bone formation in the defect area, inducing in situ remodeling.

However, in the O group, OTM in the alveolar defect region without bovine bone mineral grafts induced the less new bone formation and a low rate of tooth movement, which might have been caused by the lack of the scaffold and supporting function obtained from BG material augmentation.

### Polarized M2 macrophages are involved in mechanical force-induced remodeling of bovine bone mineral grafts in the alveolar bone defect during OTM

**Fig. 3:**
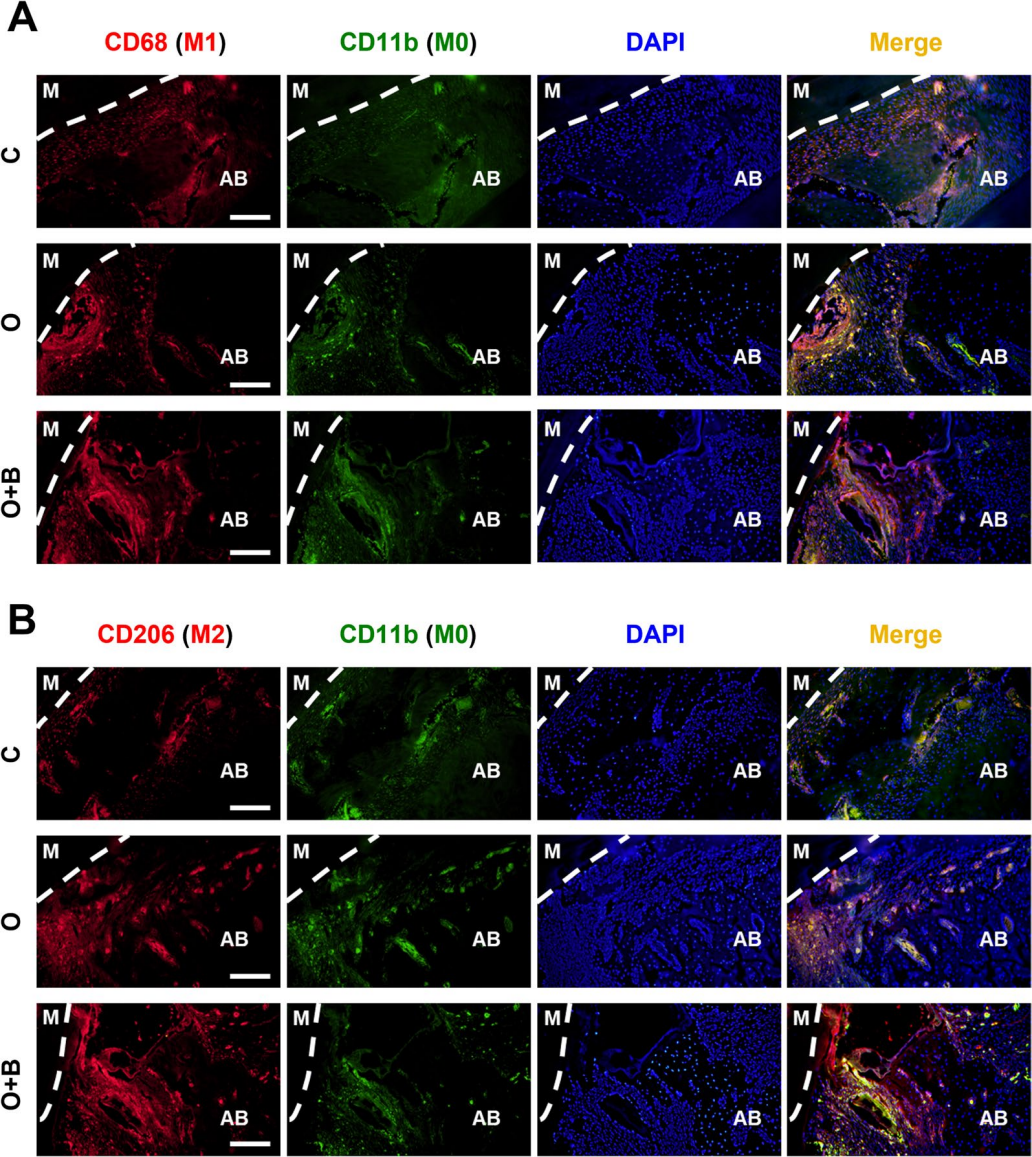
Immunofluorescence staining of markers for M1/M2 polarized macrophages in rat alveolar defects with/without BG material grafts after OTM. **A** CD68^+^CD11b^+^ labeling of M1 macrophages in each group. C: the normal control group. O: the OTM group. O + B: the OTM + BG material group. M: mesial root. AB: alveolar bone. **B** CD206^+^CD11b^+^ labeling of M2 macrophages in each group

To further clarify what type of cells were involved in the mechanical force-induced remodeling of the BG materials during OTM, IF staining was used to investigate the changes in macrophage polarization. In the O group, there were significantly increased populations of M1 macrophages with CD68^+^CD11b^+^ surface markers in the alveolar bone defect regions without bovine bone mineral grafts, while the population of M1 macrophages did not increased in the O + B and C groups (Fig. 3A).

Moreover, in the O + B group, the population of M2 polarized macrophages were significantly increased in the alveolar bone defect augmented with BG materials (Fig. 3B). This was indicated by a significant increase in the number of CD206^+^CD11b^+^ cells adjacent to the BG material graft and the alveolar bone (Fig. 3B). However, these cells were rarely observed in the O and C groups.

Additionally, we noted that the number of polarized macrophages was higher in the O and O + B groups than in the normal control group for both M1 and M2 type macrophages.

### Mechanical force promotes phagocytosis of BG materials by macrophages

**Fig. 4:**
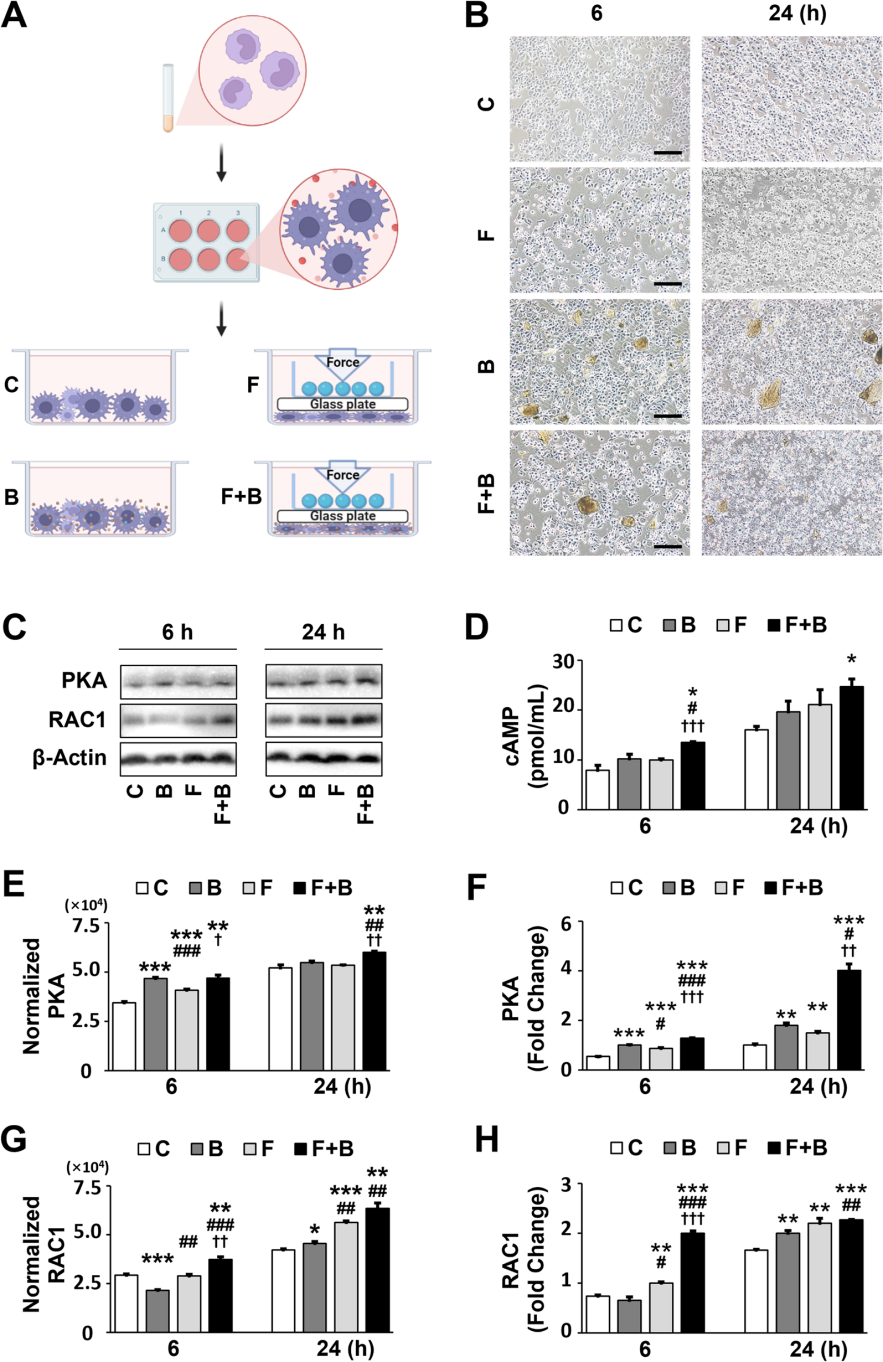
Cell culture mimicking the in vivo conditions in which macrophages in the grafts were subjected to mechanical force. **A** A schematic diagram of the cell culture study. The THP-1-induced macrophages were distributed to four groups: Control (C), BG material (B), Force (F), and Force + BG material (F + B) groups, and were cultured for 6 and 24 h, respectively. **B** Microscopic examination of cell morphology. **C** Western blotting analysis of protein levels in each group at 6 and 24 h. **D** Quantitative analysis of levels of cAMP. **E**, **F** Quantitative analysis of the protein and mRNA expression levels of PKA. **G**, **H** Quantitative analysis of the protein and mRNA expression levels of RAC1. Each value represents the mean (n = 3/group) ± the standard error. * *p* < 0.05, ** *p* < 0.01, *** *p* < 0.001, values were compared among group C and the other three groups (B, F, and F + B), respectively. ^#^ *p* < 0.05, ^##^ *p* < 0.01, ^###^ *p* < 0.001, values were compared among group B and the other two groups (F and F + B), respectively. ^†^ *p* < 0.05, ^††^ *p* < 0.01, ^†††^ *p* < 0.001, values were compared between the F and F + B groups

To explore the effects of mechanical force on the remodeling of BG materials by macrophages and the underlying molecular mechanisms, THP-1-induced macrophages were cultured with or without compressive force and BG materials for 0, 6, and 24 h. As shown in Fig. 4B, at 6 h, morphological observation revealed that there was no significant difference in the shape, size, and number of BG particles between the B and F + B groups. However, at 24 h, the BG particles were apparently smaller, and the number of material particles was decreased in the F + B group compared with that in the B group (Fig. 4B). Interestingly, no significant difference in the morphology of macrophages were observed among the C, F, B, and F + B groups at 6 and 24 h. The results suggested that mechanical force may enhance the phagocytosis and degradation of the BG particles by macrophages.

### Macrophage-mediated phagocytosis of BG materials under mechanical force is via cAMP/PKA/RAC1 signaling pathway

We further investigated the levels of cAMP (Fig. 4D), and the normalized protein and mRNA expressions of PKA, and RAC1 molecules in different groups of macrophages at 6 and 24 h (Fig. 4C, E–H). The results showed that the F + B group had the most significant increase in protein and mRNA expressions of these molecules compared with those in the other three groups at both 6 and 24 h. Specifically, the F + B group showed a significant upregulation in the levels of cAMP compared with that in all other groups (*p* < 0.05; *p* < 0.05; *p* < 0.001) at 6 h, and remained significantly increased compared with that in the C group at 24 h (*p* < 0.05) (Fig. 4D).

Likewise, the PKA protein and mRNA expression levels were significantly higher in the B, F, and F + B groups compared with that in the C group at 6 h (Fig. 4E, F; *p* < 0.001; *p* < 0.001; *p* < 0.01/*p* < 0.001). The B and F + B groups had statistically higher PKA protein levels than the F group at this time point (*p* < 0.001; *p* < 0.05). At 24 h, the F + B group had significantly higher PKA protein levels compared with those in the other three groups (Fig. 4E; all *p* < 0.01). The F + B group also showed higher *pka* mRNA expression compared with that in the B and F groups at 6 h (both *p* < 0.001) and 24 h (*p* < 0.05; *p* < 0.01) (Fig. 4F), while the F group had lower *pka* mRNA expression than the B group at 6 h (*p* < 0.05).

Finally, the F + B group had significantly higher RAC1 protein and mRNA expression levels compared with those in the other three groups at 6 h (Fig. 4G, H; *p* < 0.01/*p* < 0.001; *p* < 0.001; *p* < 0.01/*p* < 0.001). The B group showed a decrease in RAC1 protein levels compared with that in the C group (*p* < 0.001), while the F group showed an increase compared with that in the B group (*p* < 0.01) at this time point (Fig. 4G). At 24 h, the protein levels of RAC1 increased significantly in the B, F, and F + B groups compared with those in the C group (*p* < 0.05; *p* < 0.001; *p* < 0.01), with the F and F + B groups also showing significant increases compared with that in the B group (both *p* < 0.01). Additionally, the F group showed an increase in *rac1* mRNA expression compared with that in the C and B groups at 6 h (Fig. 4H; *p* < 0.01; *p* < 0.05) and all groups showed significant increases in *rac1* mRNA expression compared with that in the C group at 24 h (*p* < 0.01; *p* < 0.01; *p* < 0.001). The F + B group maintained significantly elevated *rac1* mRNA expression compared with that in the B group (*p* < 0.01) at this time point.

### Mechanical force promotes IL-4 secretion while reducing IL-1β secretion and active-MMP-9 levels in macrophages cocultured with BG materials

**Fig. 5:**
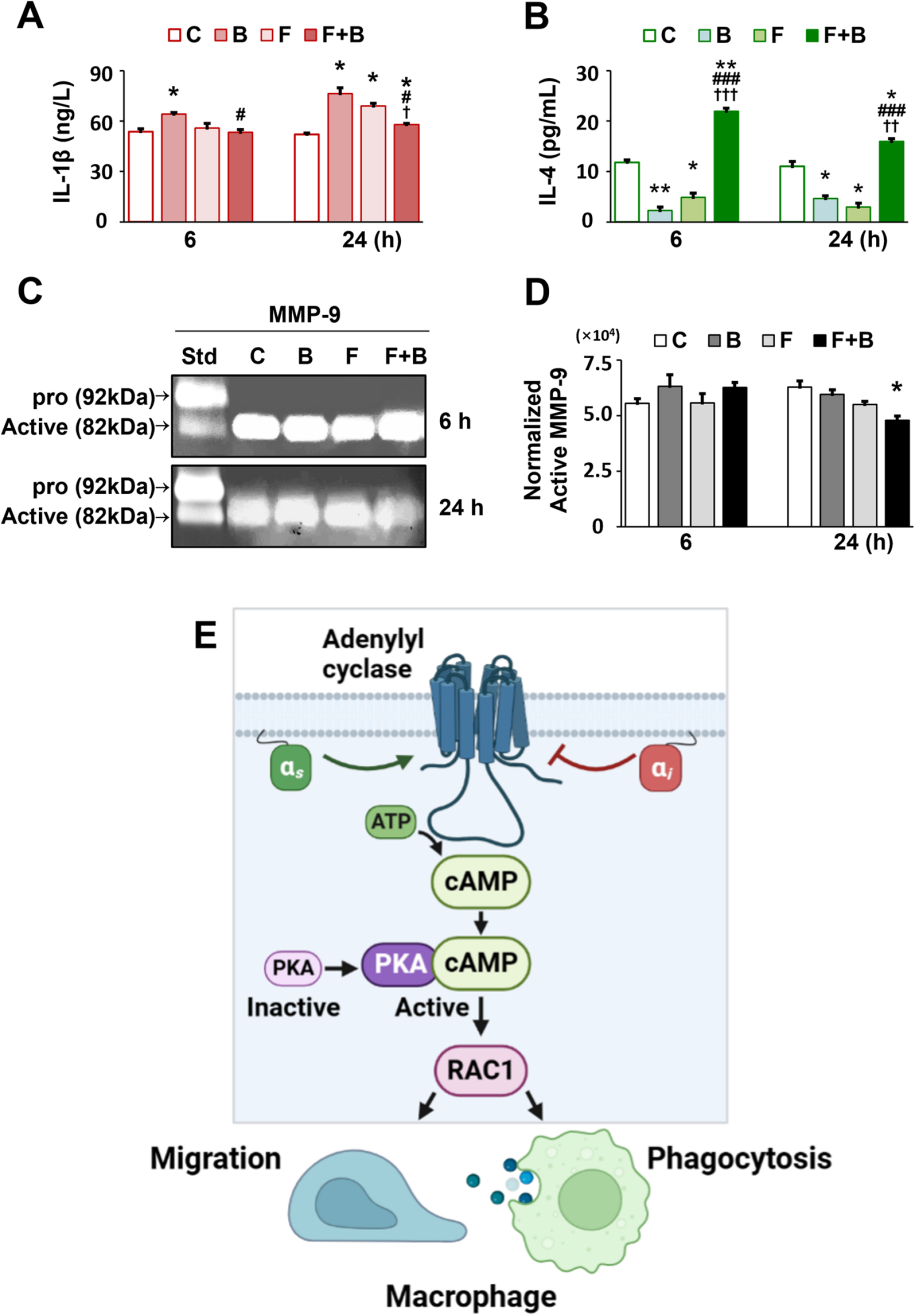
The effects of mechanical force on macrophage polarization in the presence or absence of BG materials. Assessment of IL-1β and active-MMP-9 (M1 macrophages), as well as IL-4 (M2 macrophages) levels. **A** Concentrations of IL-1β in each group at 6 and 24 h. **B** Concentrations of IL-4 in each group at 6 and 24 h. **C** Gelatin zymography detection of MMP-9 activity. **D** Quantitative analysis of active-MMP-9 levels. C: Control. B: BG material. F: Force. F + B: Force + BG material. Each value represents the mean (n = 3/group) ± the standard error. * *p* < 0.05, ** *p* < 0.01, values were compared among group C and the other three groups (B, F, and F + B), respectively. ^#^ *p* < 0.05, ^###^ *p* < 0.001, values were compared among group B and the other two groups (F and F + B), respectively. ^†^ *p* < 0.05, ^††^ *p* < 0.01, ^†††^ *p* < 0.001, values were compared between the F and F + B groups. **E** The proposed mechanism: the cAMP/PKA/RAC1 signaling pathway is involved in mechanical force-induced increase in phagocytosis and macrophage migration by promoting the polarization of M2 macrophages

To investigate the impact of mechanical force on M1/M2 macrophage polarization in the presence or absence of BG materials, IL-1β, IL-4, and active-MMP-9 were assessed (Fig. 5A–D). At 6 h, only the B group showed significantly increased IL-1β levels (Fig. 5A; *p* < 0.05), while the B, F, and F + B groups showed elevated levels of this cytokine at 24 h compared with that in the C group (all *p* < 0.05). The F + B group had the lowest levels of IL-1β compared with that in the B group at 6 h (*p* < 0.05) and compared with that in the B and F groups at 24 h (both *p* < 0.05), but the largest increase in IL-4 levels compared with those in the other three groups at both timepoints (Fig. 5B; *p* < 0.01/*p* < 0.05; *p* < 0.001; *p* < 0.001/*p* < 0.01). Conversely, both the B and F groups had markedly reduced IL-4 levels in macrophages compared with those in the C group at both 6 h (*p* < 0.01; *p* < 0.05) and 24 h (*p* < 0.05; *p* < 0.05).

Gelatin zymography was used to measure active-MMP-9 in macrophages (Fig. 5C). The F + B group had the lowest levels of active-MMP-9 at 24 h compared with that in the C group (*p* < 0.05), and showed a reduced trend of reduction compared with that in the B and F groups (Fig. 5D).

## Discussion

To date, the feasibility and safety of tooth movement into a region with bone defects augmented by BG materials has been controversial. There was a concern that the grafting material might prevent OTM by acting as a cement-like substance that adheres to both the alveolar bone and the tooth roots. However, our study found that OTM is indeed feasible and safe in these regions. This was revealed by a significant increase in the distance of OTM and more newly-formed bone in the region after grafting with bovine bone mineral materials. Additionally, previous studies also demonstrated that the augmented bone region did not obstruct OTM, nor did the grafts aggravate root resorption compared with the control group [26]. They showed that OTM immediately after bone grafting yielded the highest movement rate, which was similar to OTM performed two weeks after alveolar osteotomy alone [27].

Specifically, in the present study, the O + B group showed significant increases in four parameters, i.e., the trabecular bone volume, bone marrow volume, bone volume fraction, and trabecular thickness, indicating more bone formation in this group during OTM with BG material augmentation. This might have been caused by the recruitment of osteoclasts and inflammatory cells early in the remodeling process under mechanical force, leading to enhanced osteoclast-mediated bone resorption followed by osteoblast-mediated bone formation [6, 28, 29]. The process might accelerate the remodeling of the alveolar bone with bovine bone mineral grafts under mechanical force, resulting in more newly-formed bone tissues in the O + B group.

We also found that bovine bone mineral grafts can act as a partial support for teeth in regions with alveolar bone defects, counteracting the forces that tilt teeth toward the defect. Hence, the second molars in the O + B group were moved relatively upright without a noticeable incline from the sagittal view. Interestingly, the BG material was no longer visible in the distal area after the tooth moved past the grafting area. However, the material remained visible in the mesial area that had not yet undergone remodeling. Therefore, we hypothesized that cells in the area might participate in the biodegradation of BG particles after mechanical force-induced bone remodeling. And this hypothesis has been further confirmed by immunofluorescence (IF) staining of M1/M2 polarized macrophages as described below.

In another aspect, the development of artificial biomaterials by mimicking the extracellular matrix of bone tissue is a promising strategy for bone regeneration [30, 31]. BG particles comprise hydroxyapatite crystals with high porosity and ample surface area, which confers high compatibility and mechanical stability for the migration and adhesion of osteogenic cells [32]. Surprisingly, for the first time, we observed cells migrating to the alveolar defect region under SEM. Ultrastructurally, the BG materials were wrapped with newly-formed fibrous bone-like tissues. They served as a bridge for cells at the edges of the alveolar defect, providing a path for these cells to migrate to the BG material-augmented area, contributing to further bone repair and remodeling.

Furthermore, we identified that the polarized M2 macrophages are involved in the mechanical force-induced BG material remodeling in the alveolar defect during OTM. IF staining revealed significantly increased populations of M2 polarized macrophages in the alveolar bone defect augmented with BG material in the O + B group. Macrophages are exclusively phagocytes [33], which mainly infiltrate and respond to grafted biomaterials, and actively participate in the biodegradation of these materials [7]. Studies further determined that polarized M2 macrophages can migrate and engulf the biomaterials to promote the biodegradation and clearance of foreign substances [9, 34]. Therefore, the increased in M2 macrophages might facilitate the biodegradation of the BG material during OTM.

To explore the mechanisms by which macrophages increase phagocytosis and participate in the biodegradation of BG material, we designed a cell culture study to mimic the in vivo conditions where the macrophages in the grafts were subjected to mechanical force. The most significant increases in cAMP/PKA/RAC1 protein and mRNA expression levels was detected in the F + B group at 6 and 24 h. An increased in the cellular marker of M2 macrophages (IL-4) and a decrease in markers of M1 macrophages (IL-1β and active-MMP-9) were also determined in this group. Studies showed the cAMP/PKA pathway promotes M2 macrophage polarization [35] and increases the expression of Arginase-1/mannose receptor C-type 1 (MRC1/CD206)/ chitinase-like 3 (CHI3L1/Ym-1), which are cell surface markers of M2 macrophages [36]. Besides, RAC1 is a small G protein (~ 21 kDa) that belongs to the Rho GTPases family. It functions as a pleiotropic regulator of many cellular processes, including phagocytosis [11] and motility (through the actin network) [37]. The results of the current study suggested that mechanical force increases the phagocytosis and migration of macrophages, and promotes M2 macrophage polarization via the cAMP/PKA/RAC1 signaling pathway, leading to the biodegradation of the BG material during OTM (summarized in Fig. 5E).

## Conclusions

Bone defects grafted with bovine bone mineral grafts are feasible for OTM. The grafts serve as an internal stabilizing scaffold for macrophages to migrate to and remodel this area, maintaining the long-term stability of the osteogenic environment and facilitating the formation of a stable new bone space during OTM. In addition, the population of M2 macrophages also increased both in vivo and in cell culture, with enhanced phagocytosis via the cAMP/PKA/RAC1 pathway in response to BG particles under mechanical force.

## Figures and Tables

**Fig. 6:**
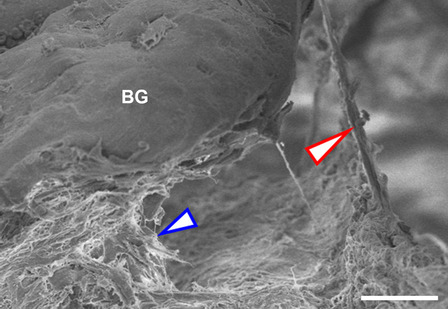
Ultrastructure of the mesial root of the 2nd molar in the alveolar defects with BG augmentation. Magnification (700 ×) of the alveolar defect area adjacent to the mesial surface (M) of the 2nd molar with BG materials augmentation. The red arrow indicates a cell migrating on the fibrous bone-like tissue that connects between the BG materials and the mature alveolar bone tissue. The blue arrow indicates the mature alveolar bone tissue. BG: bovine bone mineral graft

## Data Availability

All other supporting information is available upon request from the corresponding author.

## Appendix

See Fig. 6.

## Abbreviations

ANOVA: Analysis of variance
B: Bovine bone mineral material
BCA: Bicinchoninic acid
BG: Bovine bone mineral graft
BG material: Bovine bone mineral grafting material
BSA: Bovine serum albumin
BV/TV: Bone volume fraction
C: Control
cAMP: Cyclic adenosine monophosphate
D: Distal surface
EDTA: Ethylenediaminetetraacetic acid
ELISA: Enzyme-linked immunosorbent assay
F: Force
F + B: Force + BG material
GAPDH: Glyceraldehyde-3-phosphate dehydrogenase
IF: Immunofluorescence
IL-1β: Interleukin-1 β
IL-4: Interleukin-4
M-CSF: Macrophage colony-stimulating factor
M: Mesial
micro-CT (μCT): Microfocus computed tomography
MMP-9: Matrix metalloproteinase-9
O + B: OTM + BG material
OTM (O): Orthodontic tooth movement
PKA: Protein kinase A
PMA: Phorbol 12-myristate 13-acetate
qRT-PCR: Quantitative real-time reverse transcription polymerase chain reaction
RAC1: Ras-related C3 botulinum toxin substrate 1
SEM: Scanning-electron-microscope
S.E.M.: Standard error of the mean
THP-1 cells: Human monocytes
WB: Western blotting
